# Associations between adverse childhood experiences and diabetes among middle-aged and older Chinese: a social-ecological perspective

**DOI:** 10.4178/epih.e2023071

**Published:** 2023-08-02

**Authors:** Siyu Zhu, Leying Hou, Jiaying Ma, Shuting Li, Weidi Sun, Wen Liu, Jiajun Hao, Wenhan Xiao, Siqing Cheng, Dexing Zhang, Dong Zhao, Peige Song

**Affiliations:** 1 School of Public Health and Women’s Hospital, Zhejiang University School of Medicine, Hangzhou, China; 2 The Fourth Affiliated Hospital, International Institutes of Medicine, Zhejiang University School of Medicine, Hangzhou, China; 3 JC School of Public Health and Primary Care, The Chinese University of Hong Kong, Hong Kong, China; 4 Department of Nutrition and Food Safety, Zhejiang Provincial Center for Disease Control and Prevention, Hangzhou, China

**Keywords:** Adverse childhood experiences, Diabetes mellitus, Family

## Abstract

**OBJECTIVES:**

This study examined the associations between adverse childhood experiences (ACEs) and diabetes within a social-ecological framework, incorporating personal and environmental unfavorable conditions during childhood from family, school, and community contexts.

**METHODS:**

Data were obtained from the China Health and Retirement Longitudinal Study (2014 life history survey and 2015 survey), including 9,179 participants aged ≥45 years. ACEs were collected through self-report questionnaires, and participants were categorized based on the number of distinct ACEs experienced (0, 1, 2, 3, or ≥4 ACEs). Diabetes was defined by biomarkers, self-reported diagnosis, and treatment status. Logistic regression was conducted to explore the associations between ACEs and diabetes. Subgroup analyses were conducted by gender, age, and obesity status.

**RESULTS:**

Compared with participants without ACEs, those exposed to any ACE (odds ratio [OR], 1.19; 95% confidence interval [CI], 1.01 to 1.40), 3 ACEs (OR, 1.32; 95% CI, 1.07 to 1.62) and ≥4 ACEs (OR, 1.29; 95% CI, 1.07 to 1.56) had an increased risk of diabetes. For each additional ACE, the risk of diabetes increased by about 5%. Regarding the source of ACEs, those originating from the family (OR, 1.23; 95% CI, 1.08 to 1.41) were associated with diabetes. In terms of specific ACE types, family members with substance abuse (OR, 1.23; 95% CI, 1.01 to 1.52), emotional abuse (OR, 1.28; 95% CI, 1.12 to 1.46), and poor parental relationship (OR, 1.25; 95% CI, 1.09 to 1.43) were associated with diabetes.

**CONCLUSIONS:**

ACEs, particularly those originating from the family, were associated with diabetes. Interventions aimed at preventing and mitigating ACEs are essential for the early prevention of diabetes.

## GRAPHICAL ABSTRACT

[Fig f3-epih-45-e2023071]

## INTRODUCTION

Diabetes is a significant global public health concern, affecting an estimated 537 million adults worldwide in 2021, of whom over 75% lived in low-income and middle-income countries [1]. The number of individuals with diabetes is projected to rise to 783 million by 2045 [1]. China has the largest number of individuals with diabetes, with a projected increase from 140 million in 2021 to 174 million in 2045 [1]. In China, the prevalence of diabetes has risen markedly from less than 1% in the 1980s to nearly 11% in 2013, and further to 12.4% in 2018 [2]. This trend, coupled with the aging population, is expected to impose an even greater burden on Chinese society, particularly for middle-aged and older adults, with diabetes and its associated complications affecting the cardiovascular system, eyes, kidneys, and nerves [3].

Adverse childhood experiences (ACEs) are traumatic events that occur during childhood [4]. These experiences have long-term influences on health conditions, such as diabetes, cardiovascular disease, cancer, and depression [5]. Prior research has predominantly examined the association between a limited set of ACEs, such as physical abuse, sexual abuse, neglect [6-9], and diabetes. However, according to the social-ecological systems theory, the development of children could be influenced by multiple levels of the surrounding environment [10]. The scope of ACEs has since expanded to encompass a diverse range of experiences, such as peer and community violence [4], and emerging evidence has linked childhood poverty [11] with diabetes, further supporting the concept of ACEs.

Previous research on the associations between ACEs and diabetes has rarely examined personal and environmental ACEs simultaneously, leaving the influence of a broader concept of ACEs unseen. A social-ecological approach can be useful in understanding the complex adversities a child may encounter. To comprehensively capture the influence of ACEs on diabetes, it is, therefore, necessary to evaluate the health consequences of ACEs from a social-ecological perspective.

To address the existing research gap, this study aimed to examine the associations between ACEs within a social-ecological framework, encompassing personal and environmental unfavorable conditions from family, school, and community, and diabetes among Chinese middle-aged and older adults. This study hypothesized that (1) adults with a history of ACEs have a higher risk of diabetes; (2) the strengths of associations between ACEs and diabetes in adulthood are influenced by the number of ACEs experienced; (3) the associations between ACEs and diabetes vary according to the types of ACEs originating from different sources.

## MATERIALS AND METHODS

### Study design and population

This study used data from the China Health and Retirement Longitudinal Study (CHARLS), a nationwide longitudinal survey of adults aged ≥ 45 years in China. Detailed information on CHARLS has been described elsewhere [12,13]. Briefly, the baseline survey was conducted from June 2011 to March 2012. Through a multistage probability-proportional-to-size sampling strategy, 17,708 participants from 10,257 households in 450 villages/urban communities in 28 provinces took part in the CHARLS baseline survey. The overall household response rate was 80.5%. After the baseline survey, 2-year follow-up surveys were conducted in 2013, 2015, and 2018 with new participants included in each survey. Information on life history including experiences in childhood was additionally collected from self-reports in 2014 by trained staff. This cross-sectional study utilized data from the 2014 life history survey and the 2015 follow-up survey, as the 2015 survey provided the most recent biomarker and blood data related to diabetes among all follow-up surveys [13].

### Adverse childhood experiences

**Figure 1.**
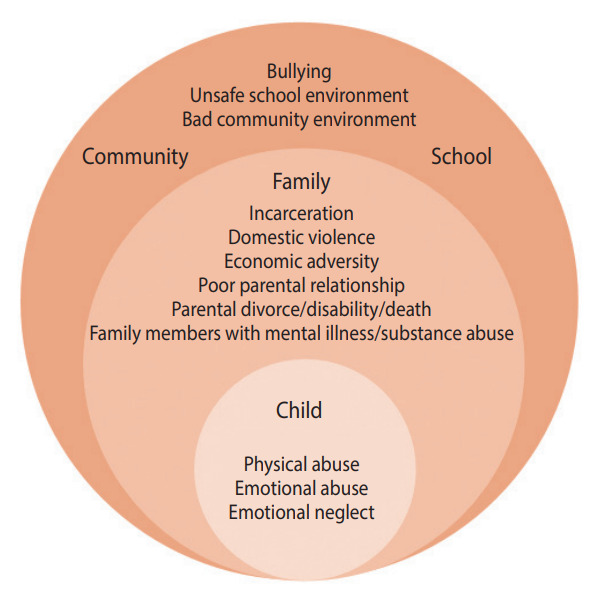
The concept of adverse childhood experiences.

The concept of ACEs based on social-ecological system theory is shown in Figure 1. From the perspectives of both personal and environmental unfavorable conditions, we broadly defined 15 types of ACEs before the age of 17 in this study: (1) personal: emotional neglect, emotional abuse, or physical abuse; (2) environmental: family (family members with mental illness, family members with substance abuse, incarceration, domestic violence, poor parental relationship, parental divorce, parental disability, parental death, or economic adversity); school (unsafe school environment or bullying); community (bad community environment or bullying). Based on the above conditions, we classified 3 sources of ACEs: children, families, communities, and schools. We then dichotomized participants based on whether they had experienced ACEs from these different sources. A detailed definition of each ACE is provided in Supplementary Material 1.

We calculated a cumulative score, referred to as “continuous ACEs,” based on the number of ACE types experienced by each individual, to measure the influence of each additional ACE on diabetes risk. The score was then categorized into 5 groups for analysis: 0, 1, 2, 3, or ≥ 4 ACEs, which were defined as the “number of ACEs.” Additionally, we dichotomized all participants based on whether they had experienced any type of ACEs.

### Definition of diabetes

Skilled nurses collected the venous blood samples. The samples were then promptly transported to the local laboratory and stored at 4°C. After being centrifuged, blood samples were stored at -20°C before being transported to a central laboratory in Beijing, where they were frozen at -80°C before analysis. Enzyme colorimetric test was used to measure the levels of glucose, total cholesterol (TC), triglycerides (TG), low-density lipoprotein cholesterol (LDL-C), and high-density lipoprotein cholesterol (HDL-C). Boronate affinity high-performance liquid chromatography was performed to measure glycated hemoglobin (HbA1c) levels. These laboratories had been recognized with the standardized certification.

According to the American Diabetes Association [14], diabetes was defined if any of the following criteria were met: (1) fasting plasma glucose ≥ 7.0 mmol/L (126 mg/dL) or random plasma glucose ≥11.1 mmol/L (200 mg/dL); (2) HbA1c ≥6.5% (48 mmol/mol); (3) self-report of a doctor’s diagnosis; (4) receiving related treatment including taking medicine or insulin injections.

### Measurements of other variables

Trained staff performed face-to-face interviews to collect information on participants’ demographic characteristics. Demographic factors included age (≥ 45 years), gender (men and women), residence (rural and urban), and educational attainment (primary school or less, middle school, high school or higher). The natural logarithm of per capita expenditure (ln[PCE]) was used to determine the economic status of households [15,16]. To represent the range of household economic levels, the bottom, middle, and top tertiles of ln(PCE) were utilized to denote low, middle, and high economic levels, respectively. Lifestyle factors included smoking status (never smoker, ex-smoker, and current smoker) and drinking status (never drinker, ex-drinker, and current drinker).

Health history included a history of hypertension and dyslipidemia. Systolic blood pressure (SBP, mmHg) and diastolic blood pressure (DBP, mmHg) were measured with an accuracy of 1 mmHg by taking 3 of blood pressure measurements on the left arm using an Omron HEM-7200 monitor (Omron Healthcare Inc., Dalian, China) when the participants were seated, relaxed, and quiet. Hypertension was defined as either a combination of (1) a self-reported diagnosis of hypertension; (2) an average SBP ≥ 140 mmHg or an average DBP ≥ 90 mmHg; or (3) use of medicine or other treatment for hypertension [17]. Dyslipidemia was defined as meeting any of the following criteria: (1) a self-reported physician diagnosis of dyslipidemia; (2) TC ≥ 240 mg/dL, LDL‐C ≥ 160 mg/dL, HDL‐C < 40 mg/dL or TG ≥ 200 mg/dL; or (3) use of lipid‐lowering medication [18].

Height was measured using a stadiometer when participants were barefoot and holding a specific position, accurate to the nearest 0.1 cm. Weight was assessed utilizing the Omron HN-286 scale (Omron Healthcare Inc.), with precision to the nearest 0.1 kg. Participants were asked to remove shoes, heavy coats and any heavy objects. Body mass index (BMI) was calculated as weight divided by the square of height (kg/m^2^) classified as normal weight (BMI < 24 kg/m^2^, including underweight) or overweight or obesity (BMI ≥ 24 kg/m^2^). Waist circumference (WC) was measured with a soft tape around the waist at the level of the navel, and was accurate to the nearest 0.1 cm. A WC of ≥ 90 cm in men and ≥ 85 cm in women was considered as central obesity [19]. Additionally, values of variables that did not conform to physiological characteristics were considered as outliers and were then excluded.

### Statistical analysis

Descriptive statistics were presented as means with standard deviation or medians with interquartile ranges (IQRs) for continuous variables with normal distributions or skewed distributions, respectively. Categorical variables were expressed as numbers with percent age (%). The characteristics of participants with or without diabetes were compared using the Wilcoxon rank sum tests for continuous variables and the chi-square test for categorical variables.

Logistic regression models were developed to assess the associations of different numbers and types of ACEs with diabetes [20]. Adjusted covariates included age, gender, residence, educational attainment, smoking status, drinking status, household economic levels, BMI, WC, and history of hypertension and dyslipidemia. Four further subgroup analyses were conducted for participants classified by gender (men and women), age groups (45-59 and ≥ 60 years), BMI groups (normal weight and overweight/obesity), and central obesity status. The adjustment for the models in the subgroup analyses varied depending on the characteristics of the population.

All statistical analyses were performed using SAS version 9.4 (SAS Institute Inc., Cary, NC, USA). The significance level was 0.05.

### Ethics statement

Informed consent was obtained from all participants, and all procedures were approved by the Ethics Review Committee of Peking University (No. IRB00001052-11014 and IRB00001052-11015).

## RESULTS

### Characteristics of participants

**Figure 2.**
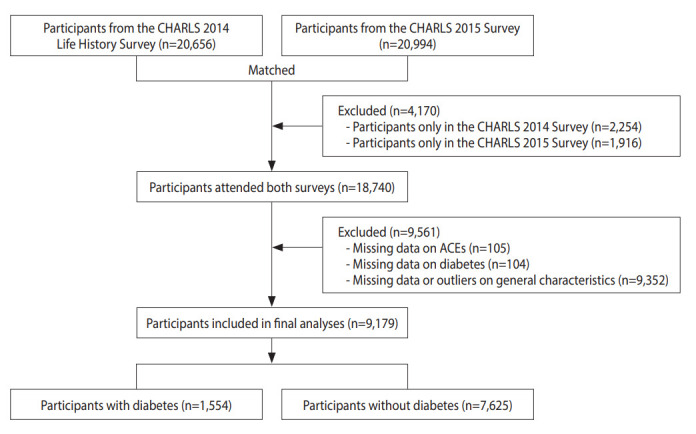
Flow chart. General characteristics include age, gender, residence, education attainment, smoking status, drinking status, household economic levels, body mass index, waist circumference, history of hypertension and dyslipidemia. ACEs, adverse childhood experiences; CHARLS, China Health and Retirement Longitudinal Study.

Attending the 2014 life history survey and the 2015 follow-up survey, a total of 9,179 participants aged 45 years and above were included in the final analyses (Figure 2). Supplementary Material 2 presents a comparison of the general characteristics of excluded and included participants. The characteristics of the included participants were stratified by diabetes status and presented in Table 1. In total, 1,554 (16.9%) participants had diabetes. The median age of participants was 59.0 years (IQR, 52.0-66.0). Of the participants, 48.0% were men. Most participants (62.7%) resided in rural areas. More than half of the participants had only completed primary school or less (58.9%) and did not smoke (57.2%) or drink (63.5%). Nearly half of the participants had central obesity (48.7%). Most participants had ACEs (85.6%), and 2,508 (27.3%) participants even experienced ≥ 4 ACEs. Significant differences were found between participants with and without diabetes in age, gender, residence, smoking status, drinking status, household economic levels, BMI, WC, BMI groups, central obesity status, history of hypertension and dyslipidemia, and continuous ACEs.

### Number of adverse childhood experiences and diabetes

The primary analysis exploring the association between ACEs and diabetes among all participants adjusted for age, gender, residence, educational attainment, smoking status, drinking status, household economic levels, BMI, WC, history of hypertension, and dyslipidemia. The results showed that participants exposed to any ACE were 1.19 times more likely to have diabetes (odds ratio [OR], 1.19; 95% confidence interval [CI], 1.01 to 1.40) than those without ACEs. Additionally, participants who had experienced 3 ACEs (OR, 1.32; 95% CI, 1.07 to 1.62) and those with ≥ 4 ACEs (OR, 1.29; 95% CI, 1.07 to 1.56) had significantly higher ORs of diabetes than those without ACEs. Furthermore, continuous ACEs were significantly associated with an increased OR of diabetes (OR, 1.05; 95% CI, 1.02 to 1.09).

A subgroup analysis categorized by gender indicated that ACEs were solely associated with a higher OR of diabetes in men. The following ACE categories were statistically significant: continuous ACEs (OR, 1.08; 95% CI, 1.03 to 1.13), 3 ACEs (OR, 1.50; 95% CI, 1.10 to 2.05), and ≥ 4 ACEs (OR, 1.41; 95% CI, 1.05 to 1.89). The subgroup analysis by age groups revealed the association between continuous ACEs and an elevated OR of diabetes in both age groups. Those aged 45-59 years with ≥ 4 ACEs and those 60 years and above with 3 ACEs had higher ORs of diabetes. When sub-grouped by whether participants had overweight/obesity, any ACE, continuous ACEs, including 2, 3, and ≥ 4 ACEs, were statistically significant with diabetes in individuals with overweight/obesity. These similar findings (except for 2 ACEs) were also observed in individuals with central obesity. More details are shown in Table 2.

### Specific types of adverse childhood experiences and diabetes

The main results of the associations between 15 types of ACEs and diabetes in all participants are shown in Table 3. Three types of ACEs including family members with substance abuse (OR, 1.23; 95% CI, 1.01 to 1.52), emotional abuse (OR, 1.28; 95% CI, 1.12 to 1.46), and poor parental relationship (OR, 1.25; 95% CI, 1.09 to 1.43) were statistically significantly associated with diabetes. Furthermore, ACEs from the family were associated with diabetes (OR, 1.23; 95% CI, 1.08 to 1.41).

In the subgroup analyses (Table 3), experiences of emotional abuse (OR, 1.42; 95% CI, 1.17 to 1.72), domestic violence (OR, 1.43; 95% CI, 1.06 to 1.93), poor parental relationship (OR, 1.41; 95% CI, 1.16 to 1.71) and ACEs from the family (OR, 1.30; 95% CI, 1.06 to 1.58), the children (OR, 1.20; 95% CI, 1.01 to 1.43) were significantly associated with diabetes for men. Similar results (except for ACEs from children) were found in participants with central obesity. In the age group of 45-59 years, participants exposed to family members with substance abuse (OR, 1.37; 95% CI, 1.02 to 1.83), emotional abuse (OR, 1.28; 95% CI, 1.04 to 1.58), bad community environment (OR, 1.30; 95% CI, 1.03 to 1.65) and ACEs from the family (OR, 1.27; 95% CI, 1.04 to 1.55) were more likely to have diabetes relative to participants without the corresponding types or sources of ACEs. Compared with those without the types of ACEs mentioned below, participants aged 60 years and over with emotional abuse (OR, 1.28; 95% CI, 1.07 to 1.52), poor parental relationship (OR, 1.29; 95% CI, 1.08 to 1.53) had higher odds of diabetes. Among the individuals with overweight/obesity, these with family members with substance abuse (OR, 1.38; 95% CI, 1.06 to 1.80), emotional abuse (OR, 1.36; 95% CI, 1.14 to 1.62), domestic violence (OR, 1.35; 95% CI, 1.05 to 1.74), poor parental relationship (OR, 1.40; 95% CI, 1.18 to 1.66) and ACEs from the family (OR, 1.33; 95% CI, 1.12 to 1.58) had increased odds of diabetes.

## DISCUSSION

Overall, ACEs were found to be associated with diabetes among middle-aged and older adults. Individuals who experienced 3 and ≥ 4 ACEs than those without ACEs were at an increased risk of diabetes. The following 3 types of ACEs were associated with a higher risk of diabetes: family members with substance abuse, emotional abuse, and poor parental relationship. By source, ACEs from the family were also associated with diabetes.

Numerous studies have reported an association between the number of ACEs and adult diabetes risk [21-24], but there are still inconsistencies in the results [25-27]. Our findings are consistent with the study conducted by Scott et al. [22], which found an association of 3 or more childhood adversities with negative physical conditions, including diabetes. The exact mechanisms underlying the association between ACEs and adult-onset diabetes are not yet fully understood. However, current evidence suggests that chronic stress may trigger biological reactions and negative coping mechanisms, such as overeating, thereby contributing to the development of diabetes [28]. From a biological perspective, early adversity influences the hypothalamic-pituitary-adrenal (HPA) axis’s role in the development of stress and inflammatory response, ultimately affecting an individual’s metabolic and inflammatory response to stress over time [29,30]. Chronic stress can also result in damage to the neuroendocrine system due to the increased release of glucocorticoids and catecholamines [30]. Early damage and biological changes may lead to decreased functioning in later stages of life, ultimately resulting in diabetes [24,31,32]. From the perspective of coping mechanisms, individuals exposed to ACEs may be at an increased risk of engaging in unhealthy behaviors, such as physical inactivity and heavy alcohol use, which can further elevate the odds of developing diabetes [5,24,33]. However, a study in Singapore found no association between exposure to any ACE and increased odds of diabetes [26]. Another study in China did not observe associations between ACEs and the risk of diabetes [27]. The inconsistencies in results across studies may be attributed to differences in the definitions of ACEs and diabetes measurements, as well as the covariates included in the analyses [34,35].

Previous studies have yielded inconsistent results regarding the associations between different types of ACEs and diabetes. Nevertheless, it is evident that not all ACEs have an equal impact on diabetes, and various types of ACEs may affect diabetes in distinct ways and to varying degrees [36]. It is well established that various types of ACEs relate to stressful life events. A study reported that stressful life events were associated with poor metabolic control [37], while another study identified perceived stress as a strong risk factor for type 2 diabetes mellitus (T2DM) [38]. Therefore, it is possible that stress from family members with substance abuse, emotional abuse, and poor parental relationship may play a significant role in the development of diabetes in later life. For instance, children living with parents who have drug and alcohol disorders often grow up under severe stress [39] and have higher rates of physical, mental, and behavioral problems [40]. The parental relationship, as defined by the perceived relationship with parents reported by participants, also contributes to stress, even though its definition is emotional and subjective as it captures the participants’ actual experiences and feelings. When participants perceive these relationships as fair or poor, they receive more stress. Stress activates the sympathetic nervous system, releases stress hormones, increases glucose production, inhibits insulin secretion, and elevates insulin resistance [41]. Moreover, according to the socialecological framework, the family is the most proximal social context for children [42], where their biological and mental functions develop and mature [43]. Therefore, adverse early family environments may exert the strongest influence on long-term biological systems, including the development of diabetes. Thus, the influence of ACEs from the family should never be disregarded.

Relatively few studies have conducted subgroup analyses by gender [11,44,45], age [26], and even obesity status (overweight/obesity and central obesity). There are some notable points. First, men who had witnessed domestic violence during childhood were more likely to have diabetes in this study. This finding may be attributed to the negative impact of witnessing domestic violence during childhood on men’s emotional health. A study of college students in China found that men who had witnessed domestic violence “often or every day” during childhood were more likely to use suppression as an emotional regulation strategy than men without such a history [46]. Furthermore, among men, but not women, emotional regulation or suppression was identified as a mediator between witnessing domestic violence during childhood and affective lability [46]. In contrast, positive emotions have been shown to have a protective effect against the development of diseases [47]. Second, the threshold for the influence of the number of ACEs varies at different age groups. This is likely related to the late age at the onset of T2DM; thus, only the associations of ≥ 4 ACEs with diabetes among adults aged 45-59 years and 3 ACEs with diabetes among adults aged over 60 years showed significance. However, further research is needed to investigate this association over time. Third, when examining these associations by BMI groups and central obesity status, individuals with overweight/obesity or central obesity were prone to be influenced by ACEs and had a high risk of diabetes. Following exposure to multiple ACEs, the risk of obesity increased by 46% [48]. The mechanisms linking ACEs to obesity include social disruption, changes in health behaviors (e.g., smoking, eating, exercise, and sleep), and chronic stress responses (namely, changes in the HPA axis) [48]. Furthermore, excessive weight contributes to diabetes by increasing the mass of adipose tissue and elevating the secretion of adipokines and resistins [49]. Given these facts, obesity is a key pathophysiological driver upstream of interventions to address T2DM and its associated metabolic complications, so weight loss is a good way to reduce the risk of T2DM [50]. It is crucial for clinicians to pay attention to people with overweight or obesity or central obesity who have also been exposed to ACEs. An early intervention in these individuals may reduce the impact of ACEs on diabetes.

This study has several strengths that contribute to a deeper understanding of the association between ACEs and diabetes in middle-aged and older adults in China. By examining both the cumulative impact of ACEs and the specific impact of individual types of ACEs, this study provides insights into how different numbers and types of ACEs may contribute to the development of diabetes. Additionally, by using an expanded concept of ACEs that includes personal, family, school, and community environments, the study provides a more comprehensive understanding of the social-ecological framework that may contribute to diabetes risk. Finally, by collecting both self-reported diagnoses and biomarkers from blood samples, the study is able to provide a more robust assessment of diabetes status.

We also acknowledge some limitations. The fact that the study only included individuals aged 45 years and above means that the results may not be generalizable to younger populations, and further research will be needed to assess the association between ACEs and diabetes in these populations. Second, we did not distinguish different types of diabetes in detail and did not consider some confounding factors related to ACEs and diabetes, such as diet, due to limited data in CHARLS. Third, information on ACEs was collected through self-reporting, and ACEs were events that occurred long before the interviews, which may have introduced recall bias. Finally, the lack of data on the severity and frequency of ACEs prevented us from exploring the associations between these factors and diabetes risk. Future research may benefit from incorporating such data to better understand the role of ACE severity and frequency in diabetes development.

In conclusion, the social-ecological framework used in this study is a valuable framework for understanding the complex association between ACEs and diabetes, and it provides important insights into the potential mechanisms underlying this association. It is important to consider the influence of ACEs on changes in chronic stress and health behaviors in order to provide correspondingly early assistance for increasing personal resilience. Additionally, more research is needed to examine the influence of family environments and investigate the underlying mechanisms linking ACEs to various population characteristics and diabetes. As such, policymakers and practitioners can better target people in need and reduce the negative consequences of ACEs on individuals, families, and society.

## Figures and Tables

**Figure f3-epih-45-e2023071:**
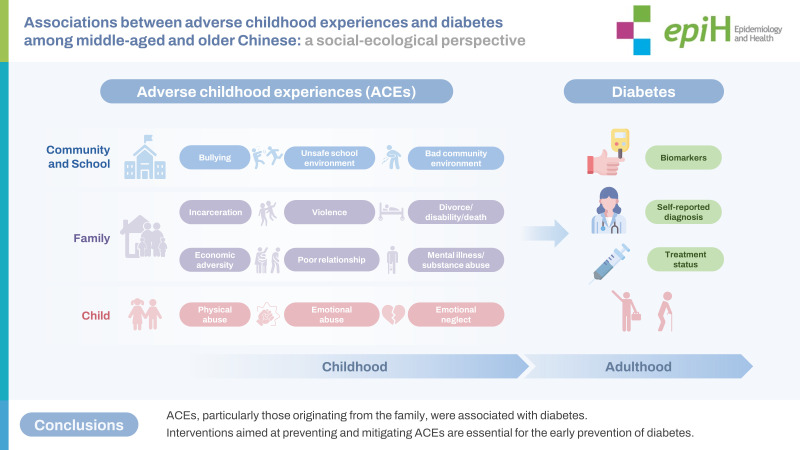


**Table 1.** Characteristics of the included participants by diabetes status

Characteristics		Total (n=9,179)	Non-diabetes (n=7,625)	Diabetes (n=1,554)	p-value^1^
Age (yr)		59.0 (52.0-66.0)	59.0 (51.0-66.0)	62.0 (54.0-68.0)	<0.001
Gender					0.004
	Men	4,409 (48.0)	3,714 (48.7)	695 (44.7)	
	Women	4,770 (52.0)	3,911 (51.3)	859 (55.3)	
Residence					<0.001
	Rural	5,752 (62.7)	4,877 (64.0)	875 (56.3)	
	Urban	3,427 (37.3)	2,748 (36.0)	679 (43.7)	
Educational attainment					0.059
	Primary school or less	5,407 (58.9)	4,459 (58.5)	948 (61.0)	
	Middle school	2,517 (27.4)	2,129 (27.9)	388 (25.0)	
	High school or higher	1,255 (13.7)	1,037 (13.6)	218 (14.0)	
Smoking status					<0.001
	Never smoker	5,249 (57.2)	4,335 (56.8)	914 (58.8)	
	Ex-smoker	1,235 (13.4)	990 (13.0)	245 (15.8)	
	Current smoker	2,695 (29.4)	2,300 (30.2)	395 (25.4)	
Drinking status					<0.001
	Never drinker	5,830 (63.5)	4,808 (63.0)	1,022 (65.8)	
	Ex-drinker	813 (8.9)	638 (8.4)	175 (11.2)	
	Current drinker	2,536 (27.6)	2,179 (28.6)	357 (23.0)	
Household economic levels^2^					<0.001
	Low	3,049 (33.2)	2,588 (33.9)	461 (29.7)	
	Middle	3,040 (33.1)	2,532 (33.2)	508 (32.7)	
	High	3,090 (33.7)	2,505 (32.9)	585 (37.6)	
WC (cm)		86.6 (79.5-94.0)	85.7 (78.9-92.7)	91.4 (84.4-98.4)	<0.001
BMI (kg/m^2^)		23.8 (21.4-26.3)	23.5 (21.2-26.0)	25.2 (22.6-27.7)	<0.001
BMI groups					<0.001
	Normal weight	4,703 (51.2)	4,153 (54.5)	550 (35.4)	
	Overweight or obesity	4,476 (48.8)	3,472 (45.5)	1,004 (64.6)	
Central obesity status					<0.001
	No	4,709 (51.3)	4,197 (55.0)	512 (33.0)	
	Yes	4,470 (48.7)	3,428 (45.0)	1,042 (67.0)	
History of hypertension					<0.001
	No	5,402 (58.9)	4,766 (62.5)	636 (40.9)	
	Yes	3,777 (41.1)	2,859 (37.5)	918 (59.1)	
History of dyslipidemia					<0.001
	No	4,498 (49.0)	3,941 (51.7)	557 (35.8)	
	Yes	4,681 (51.0)	3,684 (48.3)	997 (64.2)	
Any ACE					0.174
	No	1,324 (14.4)	1,117 (14.6)	207 (13.3)	
	Yes	7,855 (85.6)	6,508 (85.4)	1,347 (86.7)	
Continuous ACEs		2.0 (1.0-4.0)	2.0 (1.0-4.0)	2.0 (1.0-4.0)	0.016
No. of ACEs					0.087
	0	1,324 (14.4)	1,117 (14.7)	207 (13.3)	
	1	1,972 (21.5)	1,666 (21.8)	306 (19.7)	
	2	1,831 (20.0)	1,519 (19.9)	312 (20.1)	
	3	1,544 (16.8)	1,258 (16.5)	286 (18.4)	
	≥4	2,508 (27.3)	2,065 (27.1)	443 (28.5)	

Values are presented as median (interquartile range) or number (%). ACEs, adverse childhood experiences; BMI, body mass index; WC, waist circumference.

1 Statistical measurement of comparing non-diabetes and diabetes.

2 The bottom, middle, and top tertiles of the natural logarithm of per capita expenditure were utilized to denote low, middle, and high economic levels, respectively.

**Table 2.** Odds ratios and 95% confidence intervals for the association between the number of ACEs and diabetes^1^

Variables^2^		Total	Gender		Age (yr)		BMI		Central obesity status	
			Men	Women	45-59	≥60	Normal weight	Overweight or obesity	Non-central obesity	Central obesity
No. of participants		9,179	4,409	4,770	4,716	4,463	4,703	4,476	4,709	4,470
Any ACE										
	No	1.00 (reference)	1.00 (reference)	1.00 (reference)	1.00 (reference)	1.00 (reference)	1.00 (reference)	1.00 (reference)	1.00 (reference)	1.00 (reference)
	Yes	1.19 (1.01, 1.40)	1.26 (0.97, 1.64)	1.14 (0.92, 1.41)	1.22 (0.95, 1.57)	1.16 (0.92, 1.45)	1.03 (0.78, 1.35)	1.30 (1.05, 1.60)	1.10 (0.82, 1.47)	1.24 (1.01, 1.52)
Continuous ACEs		1.05 (1.02, 1.09)	1.08 (1.03, 1.13)	1.03 (0.99, 1.08)	1.06 (1.02, 1.11)	1.05 (1.00, 1.09)	1.04 (0.99, 1.09)	1.06 (1.02, 1.10)	1.06 (1.01, 1.11)	1.05 (1.01, 1.09)
No. of ACEs										
	0	1.00 (reference)	1.00 (reference)	1.00 (reference)	1.00 (reference)	1.00 (reference)	1.00 (reference)	1.00 (reference)	1.00 (reference)	1.00 (reference)
	1	1.02 (0.83, 1.24)	1.01 (0.74, 1.38)	1.03 (0.80, 1.34)	1.08 (0.80, 1.46)	0.98 (0.75, 1.28)	0.90 (0.65, 1.25)	1.09 (0.85, 1.41)	0.88 (0.62, 1.25)	1.09 (0.86, 1.39)
	2	1.16 (0.95, 1.42)	1.19 (0.88, 1.63)	1.14 (0.88, 1.49)	1.21 (0.90, 1.64)	1.11 (0.85, 1.45)	0.93 (0.67, 1.30)	1.34 (1.04, 1.72)	1.04 (0.74, 1.47)	1.23 (0.96, 1.58)
	3	1.32 (1.07, 1.62)	1.50 (1.10, 2.05)	1.18 (0.90, 1.55)	1.24 (0.91, 1.70)	1.36 (1.03, 1.78)	1.17 (0.84, 1.63)	1.40 (1.08, 1.82)	1.22 (0.86, 1.73)	1.37 (1.06, 1.77)
	≥4	1.29 (1.07, 1.56)	1.41 (1.05, 1.89)	1.21 (0.94, 1.55)	1.36 (1.02, 1.81)	1.23 (0.95, 1.58)	1.13 (0.83, 1.53)	1.39 (1.09, 1.77)	1.25 (0.91, 1.72)	1.30 (1.03, 1.65)

ACEs, adverse childhood experiences; BMI, body mass index.

1 The results from sub-grouped analyses by the corresponding factors.

2 All regressions were adjusted for age, gender (excepted in sample sub-grouped by gender), residence, educational attainment, smoking status, drinking status, household economic levels, BMI, waist circumference, history of hypertension, and dyslipidemia.

**Table 3.** Odds ratios and 95% confidence intervals for the association between types of ACEs and diabetes^1^

Variables^2^		Total	Gender		Age (yr)		BMI		Central obesity status	
			Men	Women	45-59	≥60	Normal weight	Overweight or obesity	Non-central obesity	Central obesity
Community and school		1.10 (0.97, 1.24)	1.17 (0.97, 1.40)	1.03 (0.88, 1.22)	1.09 (0.91, 1.32)	1.13 (0.96, 1.33)	1.14 (0.94, 1.38)	1.08 (0.92, 1.27)	1.20 (0.98, 1.45)	1.04 (0.89, 1.22)
	Bullying	1.13 (0.97, 1.32)	1.12 (0.90, 1.40)	1.15 (0.92, 1.43)	1.07 (0.86, 1.34)	1.22 (0.98, 1.53)	1.18 (0.92, 1.51)	1.10 (0.90, 1.35)	1.21 (0.95, 1.54)	1.08 (0.88, 1.32)
	Unsafe school environment	0.95 (0.71, 1.26)	1.02 (0.68, 1.53)	0.88 (0.59, 1.32)	0.88 (0.58, 1.33)	1.01 (0.68, 1.50)	1.19 (0.79, 1.80)	0.79 (0.53, 1.18)	1.18 (0.77, 1.80)	0.81 (0.55, 1.19)
	Bad community environment	1.05 (0.91, 1.22)	1.13 (0.90, 1.42)	0.99 (0.81, 1.21)	1.30 (1.03, 1.65)	0.95 (0.78, 1.16)	1.09 (0.87, 1.37)	1.04 (0.85, 1.27)	1.11 (0.87, 1.40)	1.03 (0.85, 1.26)
Family^3^		1.23 (1.08, 1.41)	1.30 (1.06, 1.58)	1.18 (0.98, 1.41)	1.27 (1.04, 1.55)	1.17 (0.98, 1.40)	1.08 (0.87, 1.34)	1.33 (1.12, 1.58)	1.12 (0.90, 1.40)	1.30 (1.10, 1.53)
	Incarceration	2.30 (0.95, 5.59)	3.01 (1.00, 9.11)	1.40 (0.29, 6.84)	3.14 (0.46, 21.61)	1.81 (0.66, 4.93)	2.56 (0.78, 8.40)	2.10 (0.55, 7.92)	2.73 (0.82, 9.05)	1.90 (0.52, 7.00)
	Domestic violence	1.20 (0.98, 1.46)	1.43 (1.06, 1.93)	1.03 (0.79, 1.35)	1.16 (0.85, 1.56)	1.23 (0.94, 1.60)	0.98 (0.70, 1.36)	1.35 (1.05, 1.74)	1.01 (0.72, 1.43)	1.31 (1.02, 1.68)
	Economic adversity	1.09 (0.97, 1.22)	1.01 (0.85, 1.20)	1.15 (0.98, 1.35)	1.12 (0.93, 1.34)	1.05 (0.90, 1.23)	1.00 (0.83, 1.21)	1.15 (0.99, 1.34)	0.98 (0.81, 1.19)	1.16 (1.00, 1.34)
	Poor parental relationship	1.25 (1.09, 1.43)	1.41 (1.16, 1.71)	1.13 (0.94, 1.35)	1.21 (0.98, 1.49)	1.29 (1.08, 1.53)	1.05 (0.85, 1.29)	1.40 (1.18, 1.66)	1.14 (0.92, 1.41)	1.33 (1.12, 1.57)
	Parental divorce	1.06 (0.58, 1.94)	0.89 (0.30, 2.61)	1.24 (0.59, 2.61)	1.17 (0.39, 3.51)	0.95 (0.46, 1.97)	0.98 (0.33, 2.87)	1.11 (0.53, 2.33)	0.57 (0.13, 2.42)	1.31(0.65,2.62)
	Parental disability	1.10 (0.96, 1.26)	1.12 (0.91, 1.38)	1.08 (0.90, 1.30)	1.19 (0.96, 1.46)	1.00 (0.83, 1.21)	0.99 (0.79, 1.24)	1.18 (0.98, 1.41)	1.05 (0.84, 1.32)	1.13 (0.94, 1.35)
	Parental death	0.99 (0.86, 1.15)	0.99 (0.80, 1.23)	1.00 (0.82, 1.23)	0.98 (0.76, 1.27)	1.00 (0.84, 1.20)	1.04 (0.83, 1.31)	0.96 (0.79, 1.17)	0.99 (0.78, 1.26)	0.99 (0.82, 1.20)
	Family members with mental illness	1.00 (0.87, 1.15)	1.00 (0.81, 1.23)	1.00 (0.83, 1.20)	1.01 (0.81, 1.24)	0.98 (0.82, 1.17)	1.07 (0.87, 1.33)	0.95 (0.79, 1.14)	1.02 (0.82, 1.27)	0.98 (0.82, 1.17)
	Family members with substance abuse	1.23 (1.01, 1.52)	1.21 (0.91, 1.61)	1.25 (0.92, 1.68)	1.37 (1.02, 1.83)	1.14 (0.86, 1.53)	1.06 (0.75, 1.48)	1.38 (1.06, 1.80)	1.18 (0.84, 1.65)	1.28 (0.98, 1.67)
Child		1.08 (0.96, 1.21)	1.20 (1.01, 1.43)	0.99 (0.85, 1.15)	0.99 (0.83, 1.18)	1.15 (0.98, 1.33)	1.09 (0.91, 1.31)	1.07 (0.92, 1.24)	1.19 (0.99, 1.44)	1.02 (0.88, 1.17)
	Physical abuse	1.08 (0.95, 1.23)	1.11 (0.93, 1.33)	1.05 (0.88, 1.26)	1.11 (0.92, 1.35)	1.06 (0.89, 1.26)	1.02 (0.83, 1.25)	1.12 (0.95, 1.31)	1.07 (0.87, 1.31)	1.08 (0.92, 1.27)
	Emotional abuse	1.28 (1.12, 1.46)	1.42 (1.17, 1.72)	1.16 (0.96, 1.40)	1.28 (1.04, 1.58)	1.28 (1.07, 1.52)	1.17 (0.95, 1.44)	1.36 (1.14, 1.62)	1.30 (1.05, 1.60)	1.28 (1.07, 1.52)
	Emotional neglect	0.99 (0.86, 1.15)	1.03 (0.83, 1.28)	0.96 (0.79, 1.16)	0.89 (0.71, 1.11)	1.07 (0.89, 1.30)	1.20 (0.96, 1.50)	0.87 (0.72, 1.05)	1.20 (0.95, 1.50)	0.88 (0.74, 1.06)

ACEs, adverse childhood experiences; BMI, body mass index.

1 The results from subgroup analyses by the corresponding factors.

2 All regressions were adjusted for age, gender (excepted in sample sub-grouped by gender), residence, education attainment, smoking status, drinking status, household economic levels, BMI, waist circumference, history of hypertension, and dyslipidemia.

3 The number of participants who were exposed to family member incarceration is 24, corresponding to 0.26% of the total participants.

## SUPPLEMENTARY MATERIALS

Supplement Material 1. Definition of ACEs in the CHARLS.

Supplement Material 2. Comparison of general characteristics between excluded and included participants
