# Supplementary material for: Associations between adverse childhood experiences and diabetes among middle-aged and older Chinese: a social-ecological perspective
Source: Epidemiol Health. 2023 Aug 2;45:e2023071. doi: 10.4178/epih.e2023071 (PMC10728618; doi:10.4178/epih.e2023071)
Supplement: Supplement Material 1. — Definition of ACEs in the CHARLS. [file epih-45-e2023071-Supplementary-1.docx]

**Table S1. Definition of ACEs in the CHARLS**

| **Domains of ACEs** | | **Questionnaire items** | **Answers inclusive to this domain** |
| --- | --- | --- | --- |
| Community and school | |  |  |
|  | Bullying | When you were a child, how often were you picked on or bullied by kids in your neighborhood? Is it often, sometimes, rarely, or never? | Often/Sometimes |
|  |  | When you were a child, how often were you picked on or bullied by kids in your school? Is it often, sometimes, rarely, or never? | Often/Sometimes |
|  | Unsafe school environment | When you were a child, how often did you feel worried about your physical safety at school? Is it often, sometimes, rarely, or never? | Often/Sometimes |
|  | Bad community environment | Was it safe being out alone at night in the neighborhood where you lived as a child? Is it very safe, somewhat safe, not very safe, or not safe at all? | Not very safe/Not safe at all |
|  |  | Were the neighbors of the place where you lived as a child willing to help each other out? Is it very willing to, somewhat willing to, not very willing to, or not willing to? | Not very willing to/Not willing to at all |
|  |  | Were the neighbors of the place where you lived as a child very close-knit? Is it very close-knit, somewhat close-knit, not very close-knit, or not close-knit at all? | Not very close-knit/Not close-knit at all |
| Family | |  |  |
|  | Incarceration | During the years you were growing up, which one of the followings did your male/female guardian ever have been arrested or sent to prison? | Yes |
|  | Domestic violence | Have your father/mother ever beat up your mother/father? | Often/Sometimes |
|  | Economic adversity | When you were a child before age 17, compared to the average family in the same community/village at that time, how was your family’s financial situation? | Somewhat worse off than them/A lot worse off than them |
|  | Poor parental relationship | How would you rate the relationship your parents had with each other when you were growing up? Is it excellent, very good, good, fair, or poor? | Fair/Poor |
|  | Parental divorce | Were your biological parents divorced before you were 17 years? | Yes |
|  | Parental disability | Did your male/female guardian have a long time be sick on bed when you were young? | Yes |
|  |  | Did your male/female guardian have a serious deformity when you were young? | Yes |
|  | Parental death | Did your biological father or mother die before you were 17 years? | Yes |
|  | Family members with mental illness | During the years you were growing up, had your male/female guardian showed continued signs of sadness or depression that lasted 2 weeks or more? | Yes |
|  |  | Was this problem of your male/female guardian, sadness, or depression during all, most, some, or only a little of your childhood? | All/Most |
|  |  | Did your male/female guardian have abnormality of mind when you were young? | Yes |
|  | Family members with substance abuse | During the years you were growing up, which one of the followings did your male/female guardian ever have alcoholism or drug? | Yes |
| Child | |  |  |
|  | Physical abuse | When you were growing up, did your male/female guardian ever hit you? Was that often, sometimes, rarely, or never? | Often/Sometimes |
|  | Emotional abuse | How would you rate your relationship with your male/female guardian when you were growing up? | Fair/Poor |
|  | Emotional neglect | How much love and affection did your female guardian give you while you were growing up? | Rarely/Never |

**Notes:** ACEs, adverse childhood experiences; CHARLS, China Health and Retirement Longitudinal Study.
