# Supplementary material for: Associations between adverse childhood experiences and diabetes among middle-aged and older Chinese: a social-ecological perspective
Source: Epidemiol Health. 2023 Aug 2;45:e2023071. doi: 10.4178/epih.e2023071 (PMC10728618; doi:10.4178/epih.e2023071)
Supplement: Supplement Material 2. — Comparison of general characteristics between excluded and included participants [file epih-45-e2023071-Supplementary-2.docx]

**Supplementary Material 2. Comparison of general characteristics between excluded and included participants**

| **Characteristics** | | **Total (N=18740)** | **Excluded (N=9561)** | **Included (N=9179)** | ***P* value** |
| --- | --- | --- | --- | --- | --- |
| Age (year)^a^ | | 59.0 (51.0-67.0) | 59.0 (50.0-68.0) | 59.0 (52.0-66.0) | 0.116 |
| Gender | |  |  |  | 0.019 |
|  | Men | 8704 (47.2) | 4295 (46.3) | 4409 (48.0) |  |
|  | Women | 9750 (52.8) | 4980 (53.7) | 4770 (52.0) |  |
| Residence | |  |  |  | 0.004 |
|  | Rural | 11538 (60.6) | 5786 (60.6) | 5752 (62.7) |  |
|  | Urban | 7187 (39.4) | 3760 (39.4) | 3427 (37.3) |  |
| Educational attainment | |  |  |  | <0.001 |
|  | Primary school or less | 11385 (63.1) | 5978 (63.1) | 5407 (58.9) |  |
|  | Middle school | 4824 (24.3) | 2307 (24.3) | 2517 (27.4) |  |
|  | High school or higher | 2451 (12.6) | 1196 (12.6) | 1255 (13.7) |  |
| Smoking status | |  |  |  | 0.006 |
|  | Never smoker | 10628 (59.5) | 5379 (59.5) | 5249 (57.2) |  |
|  | Ex-smoker | 2380 (12.7) | 1145 (12.7) | 1235 (13.4) |  |
|  | Current smoker | 5207 (27.8) | 2512 (27.8) | 2695 (29.4) |  |
| Drinking status | |  |  |  | <0.001 |
|  | Never drinker | 12106 (66.1) | 6276 (66.1) | 5830 (63.5) |  |
|  | Ex-drinker | 1661 (8.9) | 848 (8.9) | 813 (8.9) |  |
|  | Current drinker | 4908 (25.0) | 2372 (25.0) | 2536 (27.6) |  |
| Household economic levels | |  |  |  | <0.001 |
|  | Low | 4061 (28.1) | 1012 (28.1) | 3049 (33.2) |  |
|  | Middle | 4240 (33.4) | 1200 (33.4) | 3040 (33.1) |  |
|  | High | 4475 (38.5) | 1385 (38.5) | 3090 (33.7) |  |
| WC (cm)^a^ | | 86.0 (78.8-93.2) | 85.0 (77.4-92.4) | 86.6 (79.5-94.0) | <0.001 |
| BMI (kg/m^2^)^a^ | | 23.6 (21.2-26.2) | 23.4 (20.9-26.0) | 23.8 (21.4-26.3) | <0.001 |
| BMI groups | |  |  |  | <0.001 |
|  | Normal weight | 11668 (62.3) | 6965 (72.9) | 4703 (51.2) |  |
|  | Overweight or obesity | 7072 (37.7) | 2596 (27.1) | 4476 (48.8) |  |
| Central obesity status | |  |  |  | <0.001 |
|  | No | 11494 (62.3) | 6785 (73.1) | 4709 (51.3) |  |
|  | Yes | 6960 (37.7) | 2490 (26.9) | 4470 (48.7) |  |
| History of hypertension | |  |  |  | <0.001 |
|  | No | 11324 (60.7) | 5922 (62.5) | 5402 (58.9) |  |
|  | Yes | 7335 (39.3) | 3558 (37.5) | 3777 (41.1) |  |
| History of dyslipidemia | |  |  |  | <0.001 |
|  | No | 7537 (40.2) | 3039 (31.8) | 4498 (49.0) |  |
|  | Yes | 11203 (59.8) | 6522 (68.2) | 4681 (51.0) |  |

**Notes:** Values are presented as number (N) with percent (%). ^a^ represents medians with interquartile ranges. *P* values represent statistical measurement of comparing excluded and included participants. The bottom, middle, and top tertiles of the natural logarithm of per capita expenditure are utilized to denote low, middle, and high economic levels, respectively. BMI, body mass index; WC, waist circumference.
